# The real-world safety profile of tirzepatide: pharmacovigilance analysis of the FDA Adverse Event Reporting System (FAERS) database

**DOI:** 10.1007/s40618-024-02441-z

**Published:** 2024-08-14

**Authors:** I. Caruso, L. Di Gioia, S. Di Molfetta, M. Caporusso, A. Cignarelli, G. P. Sorice, L. Laviola, F. Giorgino

**Affiliations:** Department of Precision and Regenerative Medicine and Ionian Area, Section of Internal Medicine, Endocrinology, Andrology and Metabolic Diseases, University of Bari Aldo Moro, Bari, Italy

**Keywords:** Tirzepatide, Adverse events, GIP, Type 2 diabetes

## Abstract

**Purpose:**

Randomized controlled trials with tirzepatide (TZP) displayed unprecedented glucose and body weight lowering efficacy in individuals with type 2 diabetes and/or obesity and a safety profile similar to that of glucagon-like peptide-1 receptor agonists (GLP-1RA), mainly characterized by gastrointestinal (GI) adverse events (AE). Concerns on diabetic retinopathy, pancreato-biliary disorders, and medullary thyroid cancer were also addressed. We aimed to investigate whether the same safety issues emerged from the FDA Adverse Event Reporting System (FAERS) post-marketing surveillance database.

**Methods:**

OpenVigil 2.1-MedDRA-v24 and AERS*Mine* (data 2004Q1-2023Q3) were used to query the FAERS database. Reports of GI AE, diabetic retinopathy, pancreato-biliary disorders, and medullary thyroid cancer were investigated. The analysis was then filtered for age, gender, and designation as primary suspect. AE occurrence with TZP was compared to insulin, sodium-glucose cotransporter-2 inhibitors, metformin, and GLP-1RA.

**Results:**

Disproportionate reporting of GI [i.e., nausea (ROR 4.01, 95% CI 3.85–4.19)] and pancreato-biliary disorders [i.e., pancreatitis (ROR 3.63, 95% CI 3.15–4.19)], diabetic retinopathy (ROR 4.14, 95% CI 2.34–7.30), and medullary thyroid cancer (ROR 13.67, 95% CI 4.35–42.96) was detected. TZP exhibited a similar risk of GI AE and medullary thyroid cancer and a lower risk of most pancreato-biliary AE and diabetic retinopathy vs. GLP-1RA.

**Conclusions:**

TZP was associated with an increased risk of specific AE. However, its safety profile was similar to that of GLP-1RA, without increased risk of pancreato-biliary AE, diabetic retinopathy, and medullary thyroid cancer.

**Supplementary Information:**

The online version contains supplementary material available at 10.1007/s40618-024-02441-z.

## Introduction

Tirzepatide (TZP) is a dual glucose-dependent insulinotropic (GIP)/glucagon-like peptide-1 (GLP-1) receptor agonist approved by the Food and Drug Administration (FDA) for the management of diabetes since May 2022 and obesity since November 2023. In the latest version of the American Diabetes Association’s Standard of Medical Care in Diabetes, TZP has been listed among the most efficacious drugs for achieving glucose- and/or body weight (BW)-lowering [1]. Furthermore, a recent network meta-analysis has showed TZP to be superior to other currently available incretin-based treatment strategies in reducing HbA1c [i.e. −0.40%, 95% CI (−0.66; −0.14) vs. iDegLira] and BW [i.e. −6.56 kg, 95% CI (−7.38; −5.73) vs. semaglutide 2 mg] [2]. Randomized controlled trials (RCT) have also shown the safety profile of TZP to be similar to that of GLP-1 receptor mono-agonists (GLP-1RA) and mainly characterized by gastrointestinal (GI) adverse events (AE) [3]. Of note, both RCT [4] and real-world studies [5] with GLP-1RA have arisen concerns on other AE, including pancreato-biliary disorders, diabetic retinopathy, and thyroid (especially medullary) cancer [5], while evidence on these issues is still scarce and inconclusive for TZP. A systematic review and meta-analysis of 9 RCT showed that the administration of TZP was associated with a similar risk of pancreatitis compared to basal insulin or placebo or selected GLP-1RA, while the risk of composite gallbladder or biliary diseases vs. basal insulin or placebo was found to be increased [6]. However, a very limited number of cases of pancreato-biliary AE were reported in the 9687 patients involved in the SURPASS program [7–15]. Also, very few cases of diabetic retinopathy and no cases of medullary thyroid cancer were detected [7–15].

The FDA Adverse Event Reporting System (FAERS) database is a publicly available repository for post-marketing surveillance of FDA-approved therapies, collecting manufacturer- or customer-initiated reports of AE, product quality complaints, and medication errors as coded using the Medical Dictionary for Regulatory Activities (MedDRA) terminology. Despite intrinsic limitations, analysis of real-world data such as those collected in the FAERS database may be informative and complement the information from RCT [16]. In this study, we aimed to investigate the real-world safety profile of TZP as depicted by the FAERS database, with a particular focus on GI and pancreato-biliary AE, diabetic retinopathy, and medullary thyroid cancer.

## Research design and methods

OpenVigil 2.1-MedDRA-v24 and AERS*Mine* [17] (data 2004Q1-2023Q3) were used to query FAERS. A positive signal of disproportionality was defined as a proportional reporting ratio (PRR) of 2 or greater, chi-squared of at least 4, and 3 or more cases according to Evans’ criteria [18]. The number of total AE and of AE fulfilling Evans’ criteria was extracted for TZP vs. all other drug. Then, in agreement with the MedDRA terminology, reports of AE identified by the preferred terms (PT) under the following high level terms (HLT) were retrieved: “gastrointestinal signs and symptoms nec”, “gastrointestinal disorders nec”, “nausea and vomiting symptoms”, “dyspeptic signs and symptoms”, “diarrhoea (excl infective)”, “gastrointestinal atonic and hypomotility disorders nec”, “flatulence, bloating and distention”, “gastrointestinal and abdominal pains (excl oral and throat)”, “acute and chronic pancreatitis”, “cholecystitis and cholelithiasis”, “bile duct infections and inflammations”, “diabetic complications ophthalmic”, “thyroid neoplasms”, “thyroid disorders nec” (Supplementary Table 1). Automatic data deduplication is provided by the software and based on case and individual safety reports (ISR) numbers. The PRR and the reporting odds ratio (ROR) were extracted to assess the disproportionality reporting between cases and non-cases. The analysis of disproportionality allows to detect a higher reporting frequency of a certain adverse event with the investigated drug in comparison with a reference represented by the overall database or another specific drug [18]. Then, we repeated and restricted the search to reports indicating TZP as primary suspect drug for each AE with a positive signal of disproportionality at initial evaluation. Also, search was filtered for reports regarding female or male and adult (aged 18–65 years) or elderly (aged > 65 years) patients for all AE showing disproportionate reporting. The OpenVigil 2 × 2 contingency table (https://openvigil.pharmacology.uni-kiel.de/contingency-table-calculator.php) was used to assess difference in AE reporting according to age or gender retrieving chi-squared values with Yates’ correction, PRR and ROR. This tool was also used to assess differences in AE reporting in TZP users compared to patients on insulin ("insulin (human)" "insulin lispro" "insulin aspart" "insulin glulisine" "insulin glargine" "insulin detemir" "insulin degludec" "insulin icodec"), GLP-1RA (“exenatide”, “lixisenatide”, “liraglutide”, “albiglutide”, “semaglutide”, “dulaglutide”) as a group and individually, sodium-glucose cotransporter-2 inhibitors (SGLT-2i: “dapagliflozin”, “canagliflozin”, “empagliflozin”, “ertugliflozin”, “ipragliflozin”, “sotagliflozin”, “luseogliflozin”, “bexagliflozin”), and metformin. Concomitant use of TZP was excluded. In addition, we performed a sensitivity analysis by excluding concomitant insulin or GLP-1RA use when comparing TZP with non-insulin and non-incretin-based treatments, respectively.

Figures were created with RStudio 2023.12.1 Build 402 (MacOS, Apple Silicon version), R 4.3.3 (2024-02-29) and R package ggplot 2 version 3.4.4.

## Results

TZP was associated with a total of 20,409 reports related to 1432 AE, 116 of which fulfilled Evans’ criteria. Interestingly, 44 out of 116 (38%) AE concerned administration site and procedural complications [i.e., injection site injury, ROR 52.78, 95% confidence interval (CI) 45.75–60.89; incorrect dose administered, ROR 43.66, 95% CI 42.33–45.01], 20 (17%) GI disorders and 14 (12%) nutrition and metabolism disorders (i.e. “starvation”, ROR 41.57, 95% CI 27.22–63.48) (Supplementary Table 2).

### Tirzepatide vs. all other drugs

**Fig. 1:**
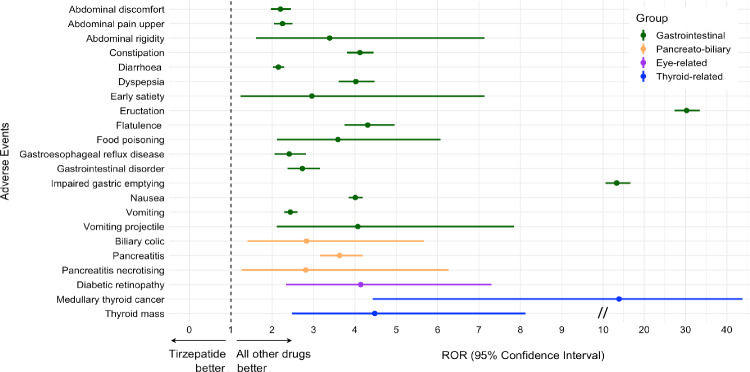
Disproportionately reported adverse events (AE) of interest for tirzepatide vs. all other drugs in the FAERS database. The forest plot shows reporting odds ratios (ROR) with 95% confidence intervals for gastrointestinal, pancreato-biliary, eye-related, and thyroid-related AE for tirzepatide vs. control drugs

With respect to the pre-specified HLT, 397 AE were classified as “gastrointestinal signs and symptoms nec”, 236 as “gastrointestinal disorders nec”, 2895 as “nausea and vomiting symptoms”, 678 as “dyspeptic signs and symptoms”, 1085 as “diarrhoea (excl infective)”, 890 as “gastrointestinal atonic and hypomotility disorders nec”, 361 as “flatulence, bloating and distention”, 578 as “gastrointestinal and abdominal pains (excl oral and throat)”, 218 as “acute and chronic pancreatitis”, 56 as “cholecystitis and cholelithiasis”, 9 as “bile duct infections and inflammations”, 12 as “diabetic complications ophthalmic”, 20 as “thyroid neoplasms”, and 25 as “thyroid disorders nec”. Disproportionate reporting of several adverse GI events was detected. Specifically, “abdominal discomfort” (ROR 2.20, 95% CI 1.97–2.45), “abdominal pain upper” (ROR 2.25, 95% CI 2.04–2.49), “abdominal rigidity” (ROR 3.39, 95% CI 1.61–7.13), “constipation” (ROR 4.12, 95% CI 3.81–4.45), “diarrhoea” (ROR 2.15, 95% CI 2.02–2.29), “dyspepsia” (ROR 4.02, 95% CI 3.61–4.48), “early satiety” (ROR 2.96, 95% CI 1.23–7.13), “eructation” (ROR 30.25, 95% CI 27.38–33.42), “flatulence” (ROR 4.31, 95% CI 3.75–4.96), “food poisoning” (ROR 3.59, 95% CI 2.12–6.07), “gastrooesophageal reflux” (ROR 2.41, 95% CI 2.06–2.82), “gastrointestinal disorder” (ROR 2.73, 95% CI 2.37–3.15), “impaired gastric emptying” (ROR 13.3, 95% CI 10.7–16.7), “pancreatitis” (ROR 3.63, 95% CI 3.15–4.19), “pancreatitis necrotising” (ROR 2.81, 95% CI 1.26–6.26), “biliary colic” (ROR 2.83, 95% CI 1.41–5.67), “nausea” (ROR 4.01, 95% CI 3.85–4.19), “vomiting” (ROR 2.44, 95% CI 2.29–2.61) and “vomiting projectile” (ROR 4.07, 95% CI 2.11–7.85) were all likely related to the administration of TZP according to Evans’ criteria (Fig. 1). A positive signal was also detected for “diabetic retinopathy” (ROR 4.13, 95% CI 2.34–7.29) among “diabetic complications ophthalmic”, and for “medullary thyroid cancer” (ROR 13.67, 95% CI 4.35–42.96) and “thyroid mass” (ROR 4.48, 95% CI 2.47–8.12) among “thyroid neoplasms” and “thyroid disorders nec” (Fig. 1). Such associations persisted after restricting the analysis to TZP as primary suspect, with the exception of “pancreatitis necrotising” (Supplementary Table 3). No signal for other gallbladder and biliary-related AE was found (i.e., ROR 1.69, 95% CI 1.05–2.72, for cholecystitis; ROR 1.35, 95% CI 0.98–1.87, for cholelithiasis).

### Safety of tirzepatide according to age and gender

**Fig. 2:**
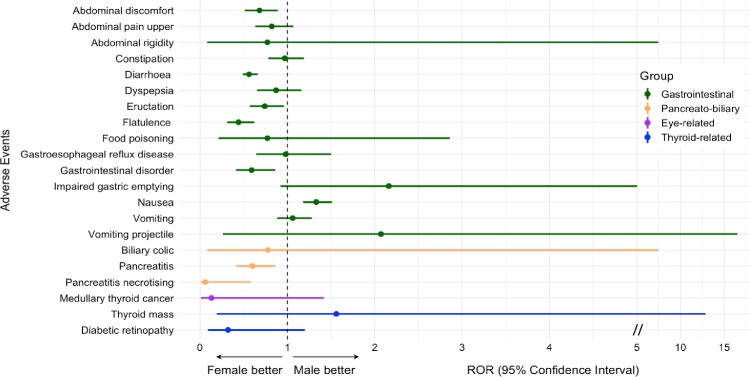
Reporting odds ratio (ROR) of adverse events (AE) of interest for tirzepatide vs. all other drugs in females vs. males. The forest plot shows ROR with 95% confidence intervals for gastrointestinal, pancreato-biliary, eye-related, and thyroid-related AE for tirzepatide vs. control drugs in females vs. males. A ROR < 1.0 indicates a disproportional lower rate of AE among reports for females

**Fig. 3:**
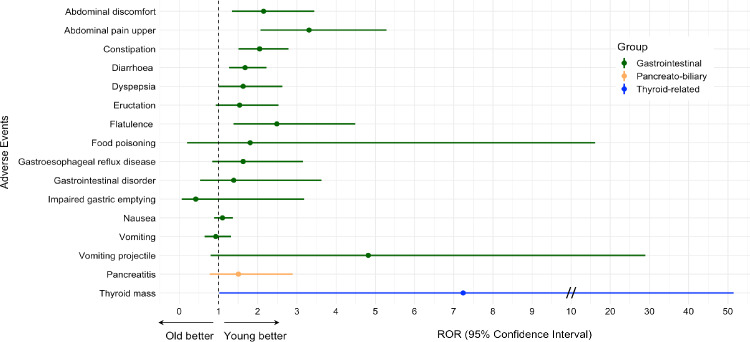
Reporting odds ratio of adverse events (AE) of interest for tirzepatide vs. all other drugs in individuals aged > 65 vs. 18–65 years. The forest plot shows reporting odds ratios (ROR) with 95% confidence intervals for gastrointestinal, pancreato-biliary, eye-related, and thyroid-related AE for tirzepatide vs. control drugs in individuals aged > 65 years (old) vs. individuals aged 18–65 years (young). A ROR < 1.0 indicates a disproportional lower rate of AE among reports for individuals aged > 65 years

A greater number of AE reports were submitted for females (13,845) than for males (3578). However, females displayed a lower risk of “abdominal discomfort”, “diarrhoea”, “eructation”, “flatulence”, “gastrointestinal disorder”, “pancreatitis”, “biliary colic” and a greater risk of “nausea” compared to males (Fig. 2). Also, a greater number of AE reports were filed for individuals aged 18–65 years (6475) rather than above 65 years (896). Nonetheless, older patients exhibited a greater risk of “abdominal discomfort”, “abdominal pain upper”, “constipation”, “diarrhoea”, “dyspepsia”, “flatulence” and “thyroid mass” occurrence (Fig. 3).

### Tirzepatide vs. GLP-1RA

**Fig. 4:**
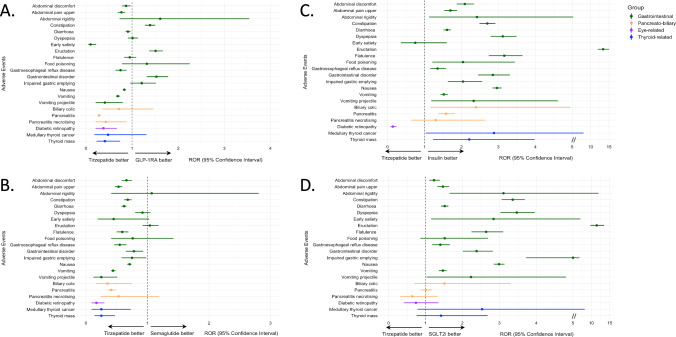
Reporting odds ratio (ROR) of adverse events (AE) of interest for tirzepatide vs. all glucagon-like peptide-1 receptor agonists (GLP-1RA, **A**), semaglutide (**B**), insulin (**C**), sodium-glucose cotransporter-2 inhibitors (SGLT-2i, **D**). The forest plot shows ROR with 95% confidence intervals for gastrointestinal, pancreato-biliary, eye-related, and thyroid-related AE for tirzepatide vs. all GLP-1RA (**A**), semaglutide (**B**), insulin (**C**), and SGLT-2i (**D**). A ROR < 1.0 indicates a disproportional lower rate of AE among reports for tirzepatide

When compared to GLP-1RA as a class, TZP displayed a lower risk of “abdominal discomfort”, “abdominal pain upper”, “gastrooesophageal reflux disease”, “nausea”, “vomiting”, “vomiting projectile”, “pancreatitis”, “pancreatitis necrotising”, “thyroid mass” and “diabetic retinopathy” and a greater risk of “constipation”, “eructation” and “gastrointestinal disorder” (Fig. 4A). The overall GI tolerability of TZP appeared to be greater than semaglutide (Fig. 4B) and worse than lixisenatide and albiglutide (Supplementary Figs. 1 and 2). Some differences in GI AE were also detected vs. exenatide, liraglutide, and dulaglutide, roughly in line with the observations regarding GLP-1RA as a class (Supplementary Figs. 3, 4, and 5). No difference in the risk of “biliary colic” was described between TZP and GLP-1RA, although TZP was associated with reduced occurrence of “cholecystitis”, “cholecystitis acute”, “cholecystitis chronic” and “cholelithiasis” (Supplementary Table 4).

TZP was associated with a lower risk of “diabetic retinopathy” vs. GLP-1RA as a class, which persisted vs. lixisenatide, liraglutide, semaglutide and dulaglutide, and following restriction of the analysis to exclude patients on concomitant insulin treatment (Fig. 4A, B; Supplementary Figs. 1, 3, 4, and 5). Also, TZP and GLP-1RA displayed a similar risk of “medullary thyroid cancer”, yet with a lower risk for TZP vs. liraglutide and semaglutide.

### Tirzepatide vs. insulin and SGLT-2i

As expected, TZP was associated with a greater risk of GI AE vs. insulin and SGLT-2i (Fig. 4C, D). Notably, no disproportionate reporting of “pancreatitis” and “biliary colic” was observed vs. SGLT-2i, while a greater risk was described vs. insulin (ROR 1.58, 95% CI 1.37–1.82, for “pancreatitis”, and ROR 2.40, 95% CI 1.16–4.94, for “biliary colic”) (Fig. 4C, D).

TZP was associated with a similar risk of “diabetic retinopathy” (ROR 0.74, 95% CI 0.41-1.35) and “medullary thyroid cancer” (ROR 2.53, 95% CI 0.78-8.21) vs. SGLT-2i. With respect to insulin, TZP was associated with a lower risk of “diabetic retinopathy” (ROR 0.14, 95% CI 0.08–0.24) and a greater risk of “medullary thyroid cancer” (ROR 2.88, 95% CI 1.37–1.82). When restricting the analysis to exclude patients on concomitant GLP-1RA, a lower risk of “diabetic retinopathy” vs. insulin persisted (ROR 0.12, 95% CI 0.06–0.22).

### Tirzepatide vs. metformin

With respect to metformin, TZP was associated with a greater risk of GI AE, a similar risk of “pancreatitis”, “pancreatitis necrotising”, “biliary colic”, “medullary thyroid cancer” and “thyroid mass” (Supplementary Fig. 6), and lower risk of “diabetic retinopathy” (ROR 0.41, 95% CI 0.23–0.72) (Supplementary Fig. 6). However, when restricting the analysis to exclude patients on concomitant insulin treatment, no difference was found between TZP and metformin in the risk of “diabetic retinopathy” (ROR 0.57, 95% CI 0.29–1.11).

## Conclusions

Real-world data from the FAERS database confirmed that treatment with TZP could increase the risk of GI AE. Disproportionate reporting for “diabetic retinopathy” and “medullary thyroid cancer” was also detected, while no signal for gallbladder and biliary-related AE was found apart from “biliary colic”.

In line with data from the SURPASS program [3], nausea, diarrhoea, and vomiting were the most commonly reported GI AE (Supplementary Table 3). Constipation, eructation, abdominal pain, and dyspepsia also emerged as frequent complaints. As expected, TZP showed a greater risk of GI AE vs. metformin, SGLT-2i and insulin, and a similar GI tolerability profile compared to GLP-1RA. Interestingly, TZP showed a lower risk of “nausea” and “vomiting” compared to GLP-1RA. This finding may have a biological explanation, as GIP receptor agonism hindered malaise and vomiting related to GLP-1 receptor agonism in three animal models [19]. GIP receptors are expressed in gamma-aminobutyric acid (GABA) ergic neurons of the area postrema of the brainstem and promote the inhibition of neurons responsible for nausea and emesis [19]. Instead, a differential effect in gastric emptying delay is unlikely to be involved, as both animal and human studies showed effects of similar magnitude for long-acting GLP-1RA and TZP [20]. Also, optimal dose and dose escalation modalities might be crucial in maximizing efficacy and tolerability of TZP, as a slower dose escalation schedule with lower initial dose and smaller subsequent dose increments improved its GI tolerability [21]. Importantly, BW reduction with TZP occurred independently of GI AE, and TZP induced a greater BW loss vs. GLP-1RA despite a similar GI tolerability [3].

Our analysis did not detect disproportionate reporting of single gallbladder and biliary-related AE with TZP except for “biliary colic”, in line with a recent meta-analysis of the SURPASS RCT program describing a significant increase in the risk of the composite of gallbladder and biliary disease only if considered as a group [6]. In addition, we found that TZP had a more favorable profile compared to GLP-1RA, which are known to be associated with an increased yet small risk of gallbladder and biliary-related disorders both in patients treated for type 2 diabetes and obesity [22]. In fact, GLP-1RA hinder gallbladder motility and emptying by reducing cholecystokinin secretion [23]. Gasbjerg et al. showed that endogenous GIP, and not GLP-1, also induced gallbladder relaxation. Dedicated studies are needed to investigate whether GIP still affects gallbladder motility in the presence of diabetes and whether these effects are replicated by TZP, as with endogenous GLP-1 and GLP-1RA [24].

Being an incretin-based medication, TZP inherited GLP-1RA-related concerns of diabetic retinopathy worsening, which peaked especially following the dissemination of the SUSTAIN-6 trial results with subcutaneous semaglutide [25]. However, this association remains controversial as meta-analyses of RCT and real-world data from the FAERS database found that GLP-1RA did not increase the risk of diabetic retinopathy outcomes vs. placebo or other glucose-lowering medications [26]. Instead, HbA1c reduction was associated with increased risk of diabetic retinopathy in a meta-regression of GLP-1RA cardiovascular outcome trials [27]. These concerns have affected the design of all subsequent trials with incretin-based medications, which in most cases excluded participants with proliferative retinopathy or retinopathy requiring immediate intervention. The ongoing FOCUS trial (NCT03811561) was also designed to assess the effects of semaglutide on diabetic retinopathy over a 5-year follow-up. Our analysis identified only 12 “diabetic retinopathy” events; TZP was associated with an increased risk of “diabetic retinopathy” compared to all other drug but with a consistently lower risk vs. GLP-1RA and insulin use, which could represent a proxy for more advanced stages of disease. A meta-analysis of the SURPASS program RCT showed a neutral effect of TZP on diabetic retinopathy, although an exiguous number of events occurred and high risk patients were excluded from these trials [28]. The role of endogenous GIP and TZP in the development of diabetic retinopathy is unknown in humans, while experiments in diabetic rats are limited to the description of GIP and GIP receptor expression in the retina [29].

The risk of medullary thyroid cancer was investigated due to the presence of a black box warning for GLP-1RA in patients at risk for this condition, despite conflicting evidence [30]. A positive signal for medullary thyroid cancer was found for TZP, although based on only 3 events. However, a higher expression of GIP receptor was found in tumor rather than normal tissue in rats and human specimens of medullary thyroid cancer, and the stimulation of GIP receptor resulted in increased secretion of calcitonin [31]. The implications of the GIP/GIP receptor axis stimulation on the occurrence of medullary thyroid cancer and patients’ prognosis remains to be elucidated.

As frequently observed with pharmacovigilance databases [32], we found that a greater number of reports of AE with TZP concerned females. This could be due to several reasons, such as a different access to healthcare services, biological differences in AE occurrence and perception between genders, and pre-existing social and structural barriers [32]. In our analysis, females appeared to be at a lower risk of some GI-related AE vs. males, although it should be acknowledged that approximately 2900 reports were not informative on patients’ gender. These findings conflict with data coming mostly from Asian studies showing that GI AE with GLP-1RA are more common in females [33] and require further confirmation in studies with TZP enrolling patients with diverse ethnicity.

Patients’ age was also underreported, as this information was missing in approximately 13,000 reports. We found that elderly patients displayed a greater risk of several GI AE and “thyroid mass”, in agreement with the notion that frailty and polypharmacy might predispose to the development of AE. Moreover, aging induces GI changes which may facilitate GI AE occurrence, and is also a well-known risk factor for cancer development [34].

The main limitations of this work are related to the intrinsic features of the FAERS database. Firstly, the FAERS includes spontaneous reports of AE without necessarily implying a cause-effect relationship with the designated primary suspect drug. Moreover, not only healthcare professionals, but also patients and their families, representatives, and even manufacturers are allowed to file AE reports. This could lead to duplicates and incomplete reporting, with clinically relevant information such as drug dose, indication for prescription and patients’ gender and age often missing or being inappropriately transmitted. AE reporting could also be affected by public awareness of certain drug-AE associations, ethnicity, geography, and timing of approval and introduction to the market [35]. Moreover, discrimination of oral or injectable semaglutide and daily or weekly exenatide was often unfeasible, preventing from assessing whether differences vs. TZP were due to the pharmacokinetic and pharmacodynamic properties of oral vs. injectable and short vs. long acting formulations, respectively. Finally, available data refer to approximately the first 16 months of TZP availability on the U.S. market, and this could at least partially account for the retrieval of a small number of less common non-GI-related AE, demanding a cautious approach to the interpretation of these findings.

In summary, real-world evidence from the FAERS database depicts a reassuring safety profile for TZP. While exhibiting impressive efficacy, TZP maintains a similar GI tolerability compared to the widely used class of GLP-1RA without increased risk of pancreatitis, diabetic retinopathy, and medullary thyroid cancer.

## Supplementary Information

Below is the link to the electronic supplementary material.

Supplementary file1 (DOCX 2588 KB)

## Data Availability

The data that support the findings of this study are available from the corresponding author, F.G., upon reasonable request.
