# Supplementary material for: The real-world safety profile of tirzepatide: pharmacovigilance analysis of the FDA Adverse Event Reporting System (FAERS) database
Source: J Endocrinol Invest. 2024 Aug 14;47(11):2671–8. doi: 10.1007/s40618-024-02441-z (PMC11473560; doi:10.1007/s40618-024-02441-z)
Supplement: Supplementary file 1 — Supplementary file1 (DOCX 2588 KB) [file 40618_2024_2441_MOESM1_ESM.docx]

**The real-world safety profile of tirzepatide: pharmacovigilance analysis of the FDA Adverse Event Reporting System (FAERS) database**

Irene Caruso^1^, Ludovico Di Gioia^1^, Sergio Di Molfetta^1^, Mariangela Caporusso^1^, Angelo Cignarelli^1^, Gian Pio Sorice^1^, Luigi Laviola^1^, Francesco Giorgino^1^

^1^Department of Precision and Regenerative Medicine and Ionian Area, Section of Internal Medicine, Endocrinology, Andrology and Metabolic Diseases, University of Bari Aldo Moro, Bari, Italy

**Supplementary files**

| Supplementary Table 1 | Pag. 1 |
| --- | --- |
| Supplementary Table 2 | Pag. 8 |
| Supplementary Table 3 | Pag. 9 |
| Supplementary Table 4 | Pag. 10 |
| Supplementary Figure 1. Reporting odds ratio of adverse events of interest for tirzepatide vs. lixisenatide | Pag. 11 |
| Supplementary Figure 2. Reporting odds ratio of adverse events of interest for tirzepatide vs. albiglutide | Pag. 12 |
| Supplementary Figure 3. Reporting odds ratio of adverse events of interest for tirzepatide vs. exenatide | Pag. 13 |
| Supplementary Figure 4. Reporting odds ratio of adverse events of interest for tirzepatide vs. liraglutide | Pag. 14 |
| Supplementary Figure 5. Reporting odds ratio of adverse events of interest for tirzepatide vs. dulaglutide | Pag. 15 |
| Supplementary Figure 6. Reporting odds ratio of adverse events of interest for tirzepatide vs. metformin | Pag. 16 |

**Table 1 Search strategy.**

| **ENDOCRINE DISORDERS** | | | | | | | | | | |
| --- | --- | --- | --- | --- | --- | --- | --- | --- | --- | --- |
| DIABETIC COMPLICATIONS | | | THYROID GLAND DISORDERS | | | | | | | |
| *Diabetic Complications Ophtalmic* | | | *Thyroid Neoplasm* | | | | | *Tyroid Disorders NEC* | | |
| Diabetic retinopathy,  Cataract diabetic,  Diabetic blindness,  Diabetic eye disease,  Diabetic glaucoma,  Diabetic retinal oedema,  Retinopathy proliferative,  Retinopathy haemorrhagic,  Retinal neovascularisation,  Diabetic keratopathy,  Diabetic ophthalmoplegia,  Non-proliferative retinopathy | | | Anaplastic thyroid cancer,  Benign neoplasm of thyroid gland,  Follicular thyroid cancer,  Medullary thyroid cancer,  Papillary thyroid cancer,  Thyroid adenoma,  Thyroid cyst,  Thyroid neoplasm,  Metastases to thyroid,  Thyroid cancer metastatic,  Hurthle cell carcinoma,  Thyroid cancer,  Thyroid cancer stage 0,  Thyroid cancer stage I,  Thyroid cancer stage II,  Thyroid cancer stage III,  Thyroid cancer stage IV,  Thyroid cancer recurrent,  Thyroid b-cell lymphoma,  Poorly differentiated thyroid carcinoma | | | | | Calcitonin secretion disorder,  Euthyroid sick syndrome,  Goitre,  Goitre congenital,  Lid lag,  Thyroid disorder,  Thyroid infarction,  Thyroid pain,  Thyroglossal cyst,  Thyroid dysfunction in pregnancy,  Thyroid mass,  Thyroid malformation,  Thyroglossal fistula,  Thyroid haemorrhage,  Thyroid fibrosis,  Ectopic thyroid,  Thyroid c-cell hyperplasia,  Hypercalcitoninaemia,  Haemorrhagic thyroid cyst,  Thyroid size decreased,  Congenital thyroid disorder,  Thyroid hemiagenesis,  Thyroid calcification,  Thyroid gland injury,  Autoimmune thyroid disorder | | |
| **HEPATOBILIARY DISORDERS** | | | | | | | | | | |
| GALLBLADDER DISORDERS | | | | | | BILE DUCT DISORDERS | | | | |
| *Cholecystitis and cholelithiasis* | | | | | | *Bile duct infections and inflammations* | | | | |
| Cholecystitis,  Cholecystitis acute,  Cholecystitis chronic,  Cholelithiasis,  Cholelithiasis obstructive,  Gallstone ileus,  Emphysematous cholecystitis,  Cholecystitis infective,  Pseudocholelithiasis,  Cholelithiasis migration,  Haemorrhagic cholecystitis,  Ischaemic cholecystitis | | | | | | Biliary colic,  Cholangiolitis,  Cholangitis,  Cholangitis acute,  Cholangitis sclerosing,  Cholecystocholangitis,  Biliary sepsis,  Biliary tract infection,  Cholangitis chronic,  Biliary abscess,  Biliary tract infection viral,  Biliary tract infection bacterial,  Biliary tract infection fungal,  Lemmel's syndrome,  Biliary tract infection cryptosporidial,  Duodenal papillitis,  Cholangitis infective,  Recurrent pyogenic cholangitis,  Immune-mediated cholangitis,  Autoimmune cholangitis | | | | |
| **GASTROINTESTINAL DISORDERS** | | | | | | | | | | |
| EXOCRINE PANCREAS CONDITIONS | | GASTROINTESTINAL MOTILITY AND DEFECATION CONDITIONS | | | | | | | | |
| *Acute and chronic pancreatitis* | | *Diarrhoea (excl infective)* | | | *Gastrointestinal atonic and hypomotility disorders NEC* | | | | *Dyspeptic signs and symptoms* | |
| Pancreatitis,  Pancreatitis acute,  Pancreatitis chronic,  Pancreatitis haemorrhagic,  Pancreatitis necrotising,  Pancreatitis relapsing,  Pancreatic abscess,  Cytomegalovirus pancreatitis,  Pancreas infection,  Oedematous pancreatitis,  Pancreatorenal syndrome,  Pancreatic phlegmon,  Hereditary pancreatitis,  Alcoholic pancreatitis,  Pancreatitis fungal,  Pancreatitis bacterial,  Pancreatitis viral,  Ischaemic pancreatitis,  Lupus pancreatitis,  Autoimmune pancreatitis,  Traumatic pancreatitis,  Haemorrhagic necrotic pancreatitis,  Obstructive pancreatitis,  Radiation pancreatitis,  Immune-mediated pancreatitis,  Subacute pancreatitis,  Walled-off pancreatic necrosis | | Diarrhoea,  Diarrhoea haemorrhagic,  Diarrhoea neonatal,  Post procedural diarrhoea | | | Constipation,  Gastric atony,  Gastric dilatation,  Gastroesophageal reflux disease,  Gastroparesis, Postoperative  impaired gastric emptying,  Intestinal dilatation,  Intestinal pseudo-obstruction,  Diabetic gastroparesis,  Gastrointestinal hypomotility,  Gastric hypomotility,  Autonomic failure syndrome,  Neonatal intestinal dilatation,  Infrequent bowel movements,  Hollow visceral myopathy,  Duodenogastric reflux,  Gastrooesophageal sphincter insufficiency,  Sandifer's syndrome,  Oesophageal hypomotility,  Intestinal atony,  Post procedural constipation,  Pesophageal atony,  Pyloric sphincter insufficiency,  Intestinal neuronal dysplasia,  Obstructive defaecation,  Enteric neuropathy,  Constipation neonatal | | | | Dyspepsia,  Eructation, Epigastric discomfort,  Biliary dyspepsia | |
| GASTROINTESTINAL SIGNS AND SYMPTOMS | | | | | | | | | | |
| *Flautulence bloating and distention* | *Gastrointestinal signs and symptoms NEC* | | | *Nausea and vomiting symptoms* | | | *Gastrointestinal and abdominal pains (excl oral and throat)* | | | *Gastrointestinal disorders NEC* |
| Abdominal distention,  Flatulence  aerophagia | Abdominal discomfort,  Acute abdomen,  Breath odour,  Bruxism,  Dumping syndrome,  Dysphagia, Encopresis,  Hiccups, Hyperphagia,  Incontinence,  Mastication disorder,  Merycism,  Odynophagia,  Pelvic pain,  Peristalsis visible,  Peripancreatic fluid collection,  Foetor hepaticus,  Oesophageal discomfort,  Post cholecystectomy syndrome,  Cullen's sign,  Early satiety,  Gastrocardiac syndrome,  Radiation dysphagia,  Abdominal symptom,  Hypophagia,  Pelvic discomfort,  Portal venous gas,  Oesophageal food impaction,  Intestinal congestion,  Malignant dysphagia,  Radiation sickness syndrome,  Myochosis,  Intestinal calcification,  Fixed bowel loop,  Gastrointestinal wall thickening,  Gastrointestinal wall thinning,  Bradyphagia,  Anal incontinence,  Gastrointestinal somatic symptom disorder,  White nipple sign,  Wischnewsky spots,  Pharyngeal dystonia,  Overflow diarrhoea,  Cervicogenic dysphagia,  Intra-abdominal calcification | | | Hyperemesis gravidarum,  Meniere's disease,  Morning sickness  nausea,  Retching,  Vomiting,  Vomiting projectile,  Vomiting psychogenic,  Acetonaemic vomiting, Dialysis disequilibrium syndrome,  Cyclic vomiting syndrome,  Infantile spitting up,  Acute vestibular syndrome,  Faecal vomiting,  Post-tussive vomiting,  Procedural nausea,  Procedural vomiting,  Regurgitation  infantile, Vomiting  discoloured, Vomit  cannabinoid, Hyperemesis syndrome | | | Abdominal pain,  Abdominal pain lower,  Abdominal pain upper,  Abdominal rigidity,  Abdominal tenderness,  Gastrointestinal pain,  Infantile colic,  Oesophageal pain,  Abdominal migraine,  Abdominal rebound tenderness,  Visceral pain | | | Abdominal transposition,  Bezoar,  Diabetic gastropathy,  Food poisoning,  Foreign body in mouth,  Gastrointestinal disorder,  Gastroptosis,  Pelvic mass,  Neurogenic bowel,  Intestinal mucosal hypertrophy,  Diabetic enteropathy,  Diabetic gastroenteropathy,  Gastric disorder,  Intestinal prolapse,  Pneumatosis, Intestinalis  pylorus dilatation,  Colon injury,  Appendiceal mucocoele,  Gastrointestinal aedema,  Intestine transplant rejection,  Abdominal compartment syndrome,  Intestinal mass,  Gastrointestinal amyloidosis,  Protein-losing gastroenteropathy,  Abdominal injury,  Appendix disorder,  Gastrointstinal injury,  Pelvic organ injury,  Gastrointestinal disorder congenital,  Gastrolithiasis,  Uraemic gastropathy,  Complications of intestinal transplant,  Gastrointestinal stoma complications,  Visceral oedema,  Gastrointestinal anastomotic leak  Ccute graft versus host disease in intestine,  Stomach mass,  Gastric xanthoma,  Intestinal mucosal atrophy,  Gastrointestinal mucosal exfoliation,  Intestinal anastomosis complication,  Intestinal steatosis,  HIV enteropathy,  Chemical burn of gastrointestinal tract,  Gastric mucosa erythema,  Functional gastrointestinal disorder,  Chronic graft versus host disease in intestine,  Intestinal smooth muscle hypertrophy,  Abdominal lymphadenopathy,  Appendicolith,  Gastrointestinal anastomotic complication,  Gastric prolapse,  Cascade stomach,  Graft versus host disease in gastrointestinal tract,  Burn oral cavity,  Gastrointestinal wall abnormal,  Gastric fibrosis,  Neonatal gastrointestinal disorder,  Gastrointestinal organ contusion,  Lymphoid hyperplasia of intestine,  Gastric pneumatosis,  Atypical haemolytic uraemic syndrome,  Foreign body in gastrointestinal tract  Gut fermentation syndrome,  Ipex syndrome,  Gastrointestinal procedural complication,  Dysbiosis,  Foreign body in throat,  Foreign body ingestion,  Acute graft versus host disease oral,  Chronic graft versus host disease oral,  Intestinal mucosal tear,  Intra-abdominal organ avulsion,  Intestinal lipomatosis,  Intestinal vascular disorder |

System Organ Class (SOC) is indicated in bold capital letters, High Level Group Terms (HLGT) are indicated in capital letters; High Level Terms (HLT) are indicated in italics.

NEC, not elsewhere classified.

**Supplementary Table 2. Adverse events with tirzepatide grouped according to System Organ Class**

| **SOC** | **N** | **%** |
| --- | --- | --- |
| General disorders and administration site conditions | 28 | 24.14 |
| Gastrointestinal disorders | 20 | 17.24 |
| Injury, poisoning and procedural complications | 16 | 13.80 |
| Investigations | 17 | 14.65 |
| Metabolism and nutrition disorders | 14 | 12.07 |
| Endocrine disorders | 6 | 5.17 |
| Skin and subcutaneous tissue disorders | 3 | 2.59 |
| Nervous system disorders | 2 | 1.72 |
| Product issues | 2 | 1.72 |
| Reproductive system and breast disorder | 2 | 1.72 |
| Psychiatric disorder | 1 | 0.86 |
| Hepatobiliary disorders | 1 | 0.86 |
| Ear and labyrinth disorder | 1 | 0.86 |
| Blood and lymphatic system disorder | 1 | 0.86 |
| Infections and infestations | 1 | 0.86 |
| Cardiac disorder | 1 | 0.86 |

SOC, System Organ Class; N, number of events; %, percentage

**Supplementary Table 3. Analysis restricted to tirzepatide as primary suspect**

| **Adverse event** | **N** | **Odds Ratio** | **CI 95%** |
| --- | --- | --- | --- |
| nausea | 2456 | 4.023 | 3.856, 4.197 |
| diarrhoea | 1062 | 2.145 | 2.016, 2.282 |
| vomiting | 928 | 2.460 | 2.303, 2.628 |
| constipation | 655 | 4.120 | 3.810, 4.455 |
| eructation | 409 | 30.338 | 27.441, 33.539 |
| abdominal pain upper | 377 | 2.252 | 2.033, 2.494 |
| dyspepsia | 325 | 3.996 | 3.580, 4.461 |
| abdominal discomfort | 314 | 2.168 | 1.939, 2.424 |
| flatulence | 196 | 4.325 | 3.755, 4.98 |
| pancreatitis | 188 | 3.658 | 3.167, 4.225 |
| gastrointestinal disorder | 186 | 2.752 | 2.381, 3.18 |
| gastroesophageal reflux disease | 156 | 2.408 | 2.057, 2.82 |
| impaired gastric emptying | 78 | 13.246 | 10.578, 16.585 |
| food poisoning | 14 | 3.652 | 2.159, 6.178 |
| thyroid mass | 11 | 4.566 | 2.522, 8.265 |
| diabetic retinopathy | 10 | 3.504 | 1.882, 6.525 |
| vomiting projectile | 9 | 4.147 | 2.153, 7.991 |
| biliary colic | 8 | 2.88 | 1.44, 5.77 |
| abdominal rigidity | 7 | 3.451 | 1.642, 7.256 |
| early satiety | 5 | 3.014 | 1.251, 7.258 |
| pancreatitis necrotising | 5 | 2.379 | 0.988, 5.726 |
| medullary thyroid cancer | 3 | 13.921 | 4.430, 43.749 |

CI, confidence interval; N, number of events

**Supplementary Table 4. Reporting odds ratio of adverse events identified with the HLT of “cholecystitis and cholelithiasis” for tirzepatide vs. all GLP-1RA**

| **Adverse event** | **Odds Ratio** | **CI 95%** |
| --- | --- | --- |
| cholecystitis | 0.55 | 0.35, 0.88 |
| cholecystitis acute | 0.13 | 0.04, 0.39 |
| cholecystitis chronic | 0.09 | 0.01, 0.69 |
| cholelithiasis | 0.36 | 0.26, 0.49 |
| cholelithiasis obstructive | n.a. | n.a. |
| gallstone ileus | n.a. | n.a. |
| emphysematous cholecystitis | n.a. | n.a. |
| cholecystitis infective | 0.26 | 0.06, 1.05 |
| pseudocholelithiasis | n.a. | n.a. |
| cholelithiasis migration | n.a. | n.a. |
| haemorrhagic cholecystitis | n.a. | n.a. |
| ischaemic cholecystitis | n.a. | n.a. |

CI, confidence interval; n.a., not applicable (0 adverse events detected for tirzepatide).

**Supplementary Figure 1. Reporting odds ratio of adverse events of interest for tirzepatide vs. lixisenatide.**


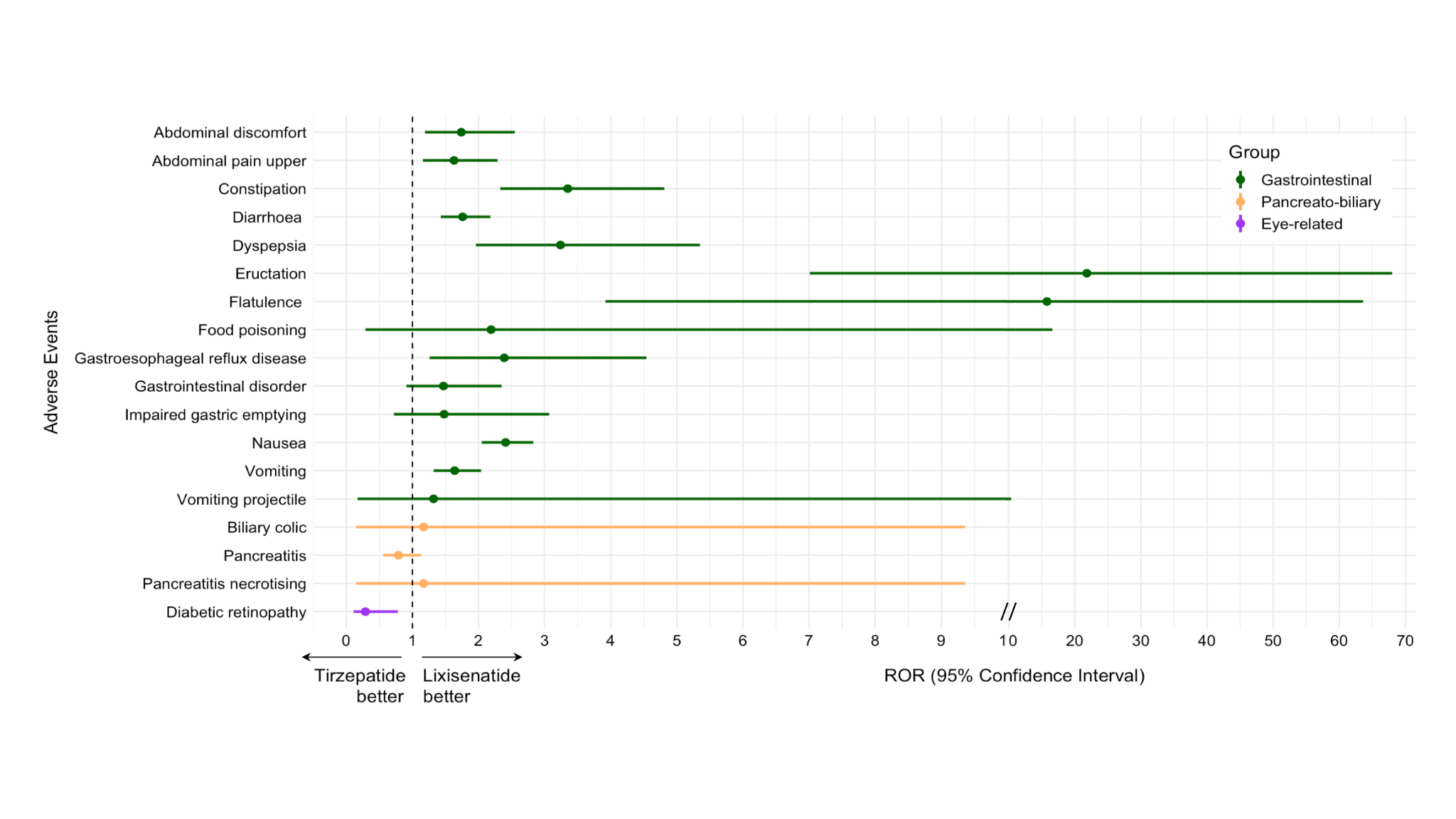
The forest plot shows reporting odds ratios (ROR) with 95% confidence intervals (CI) for gastrointestinal, pancreato-biliary, eye-related, and thyroid-related adverse events (AEs) for tirzepatide versus lixisenatide. A ROR <1.0 indicates a disproportional lower rate of AEs among reports for tirzepatide.

**Supplementary Figure 2. Reporting odds ratio of adverse events of interest for tirzepatide vs. albiglutide.**
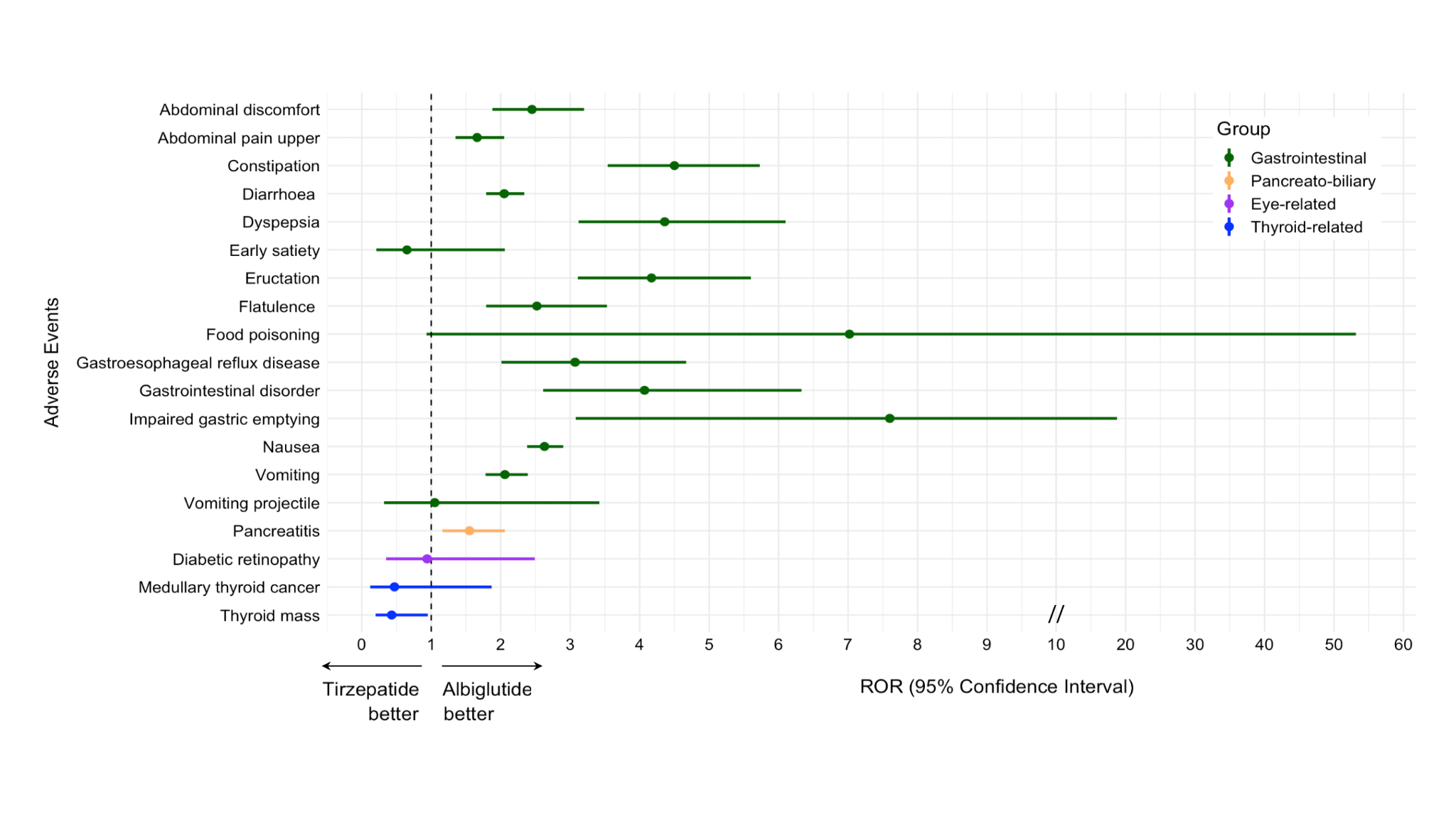
The forest plot shows reporting odds ratios (ROR) with 95% confidence intervals (CI) for gastrointestinal, pancreato-biliary, eye-related, and thyroid-related adverse events (AEs) for tirzepatide versus albiglutide. A ROR <1.0 indicates a disproportional lower rate of AEs among reports for tirzepatide.

**Supplementary Figure 3. Reporting odds ratio of adverse events of interest for tirzepatide vs. exenatide.**
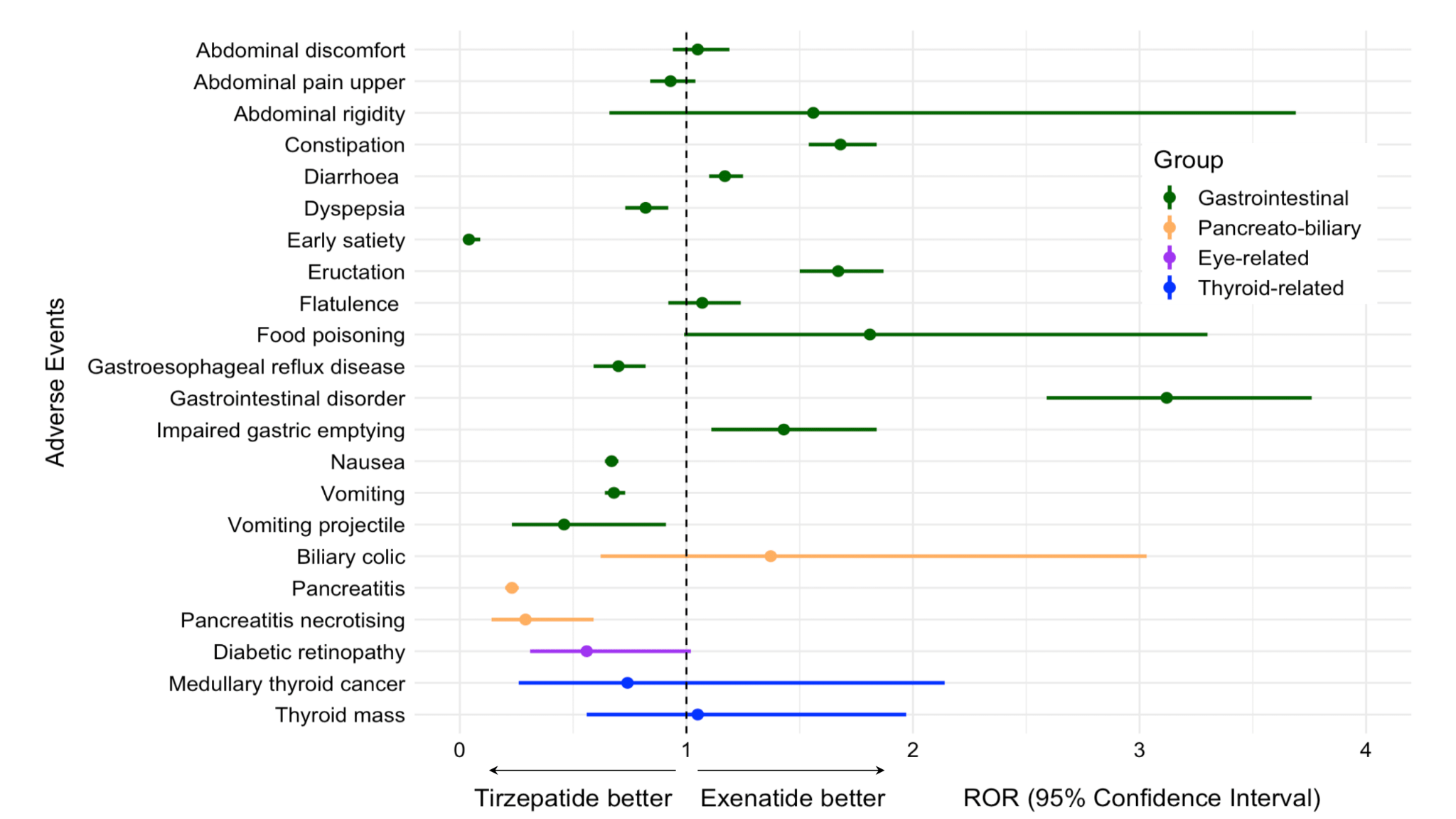
The forest plot shows reporting odds ratios (ROR) with 95% confidence intervals (CI) for gastrointestinal, pancreato-biliary, eye-related, and thyroid-related adverse events (AEs) for tirzepatide versus exenatide. A ROR <1.0 indicates a disproportional lower rate of AEs among reports for tirzepatide.

**Supplementary Figure 4. Reporting odds ratio of adverse events of interest for tirzepatide vs. liraglutide.**
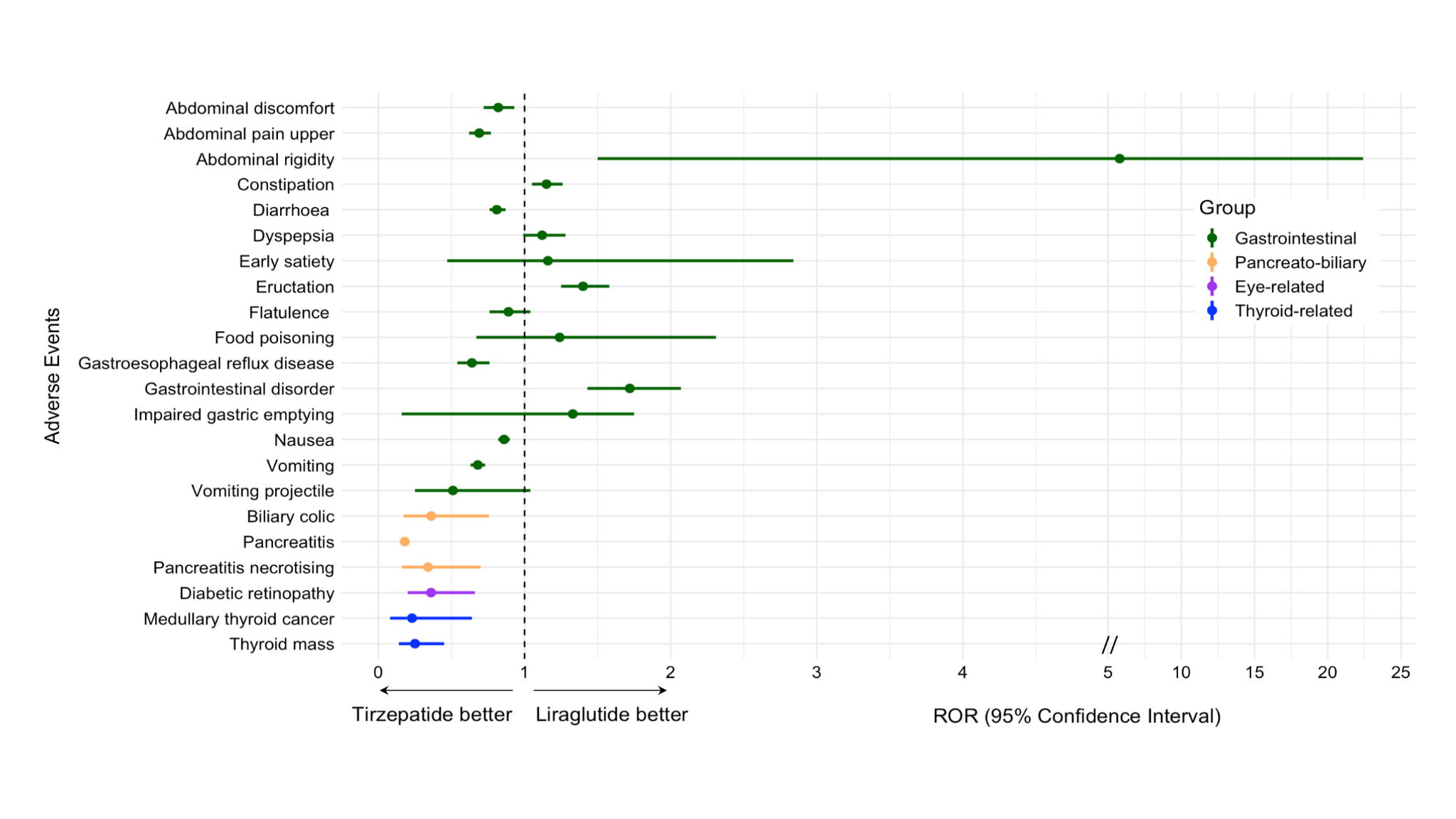
The forest plot shows reporting odds ratios (ROR) with 95% confidence intervals (CI) for gastrointestinal, pancreato-biliary, eye-related, and thyroid-related adverse events (AEs) for tirzepatide versus liraglutide. A ROR <1.0 indicates a disproportional lower rate of AEs among reports for tirzepatide.

**Supplementary Figure 5. Reporting odds ratio of adverse events of interest for tirzepatide vs. dulaglutide.
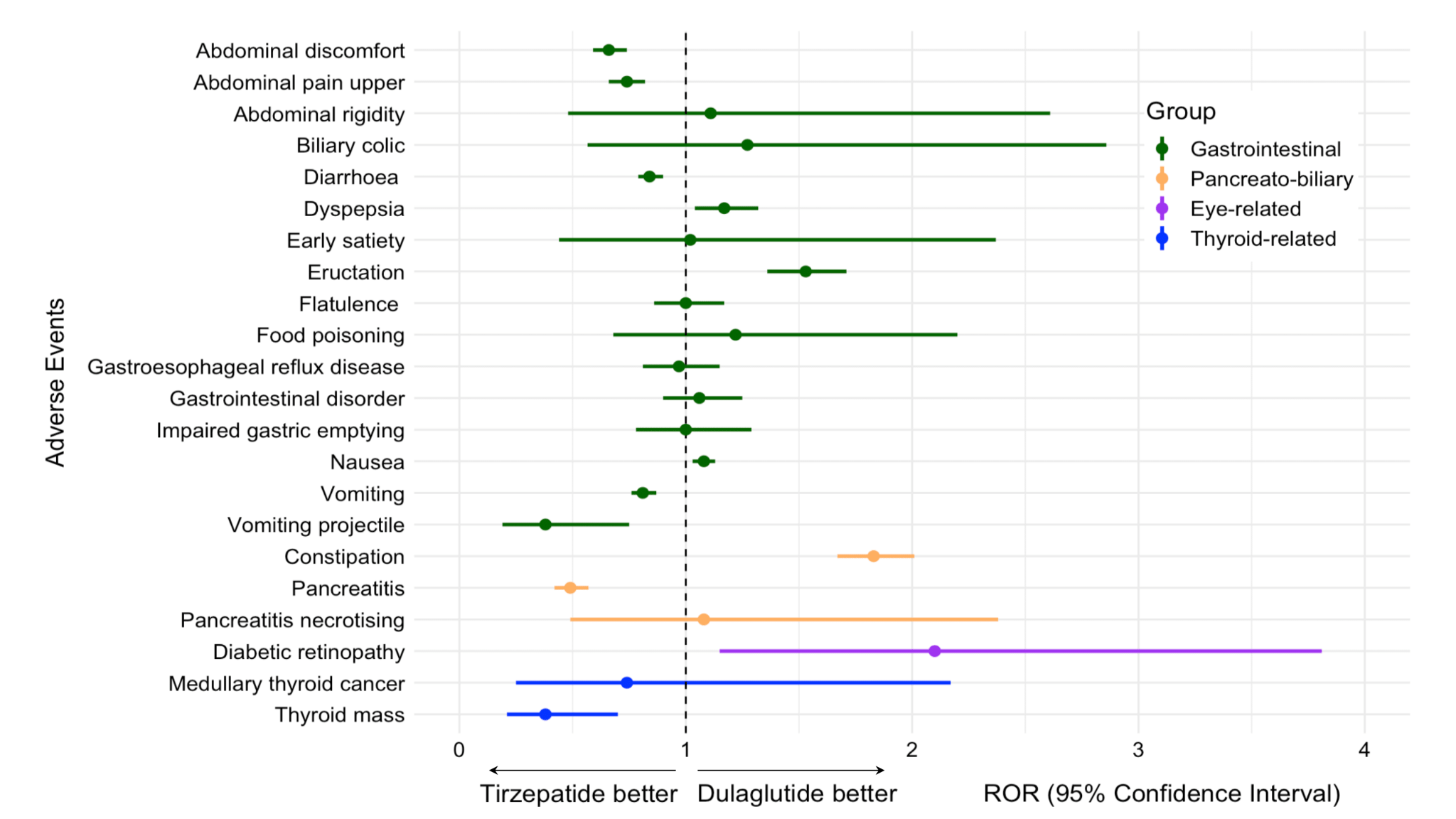
**

The forest plot shows reporting odds ratios (ROR) with 95% confidence intervals (CI) for gastrointestinal, pancreato-biliary, eye-related, and thyroid-related adverse events (AEs) for tirzepatide versus dulaglutide. A ROR <1.0 indicates a disproportional lower rate of AEs among reports for tirzepatide.

**Supplementary Figure 6. Reporting odds ratio of adverse events of interest for tirzepatide vs. metformin.
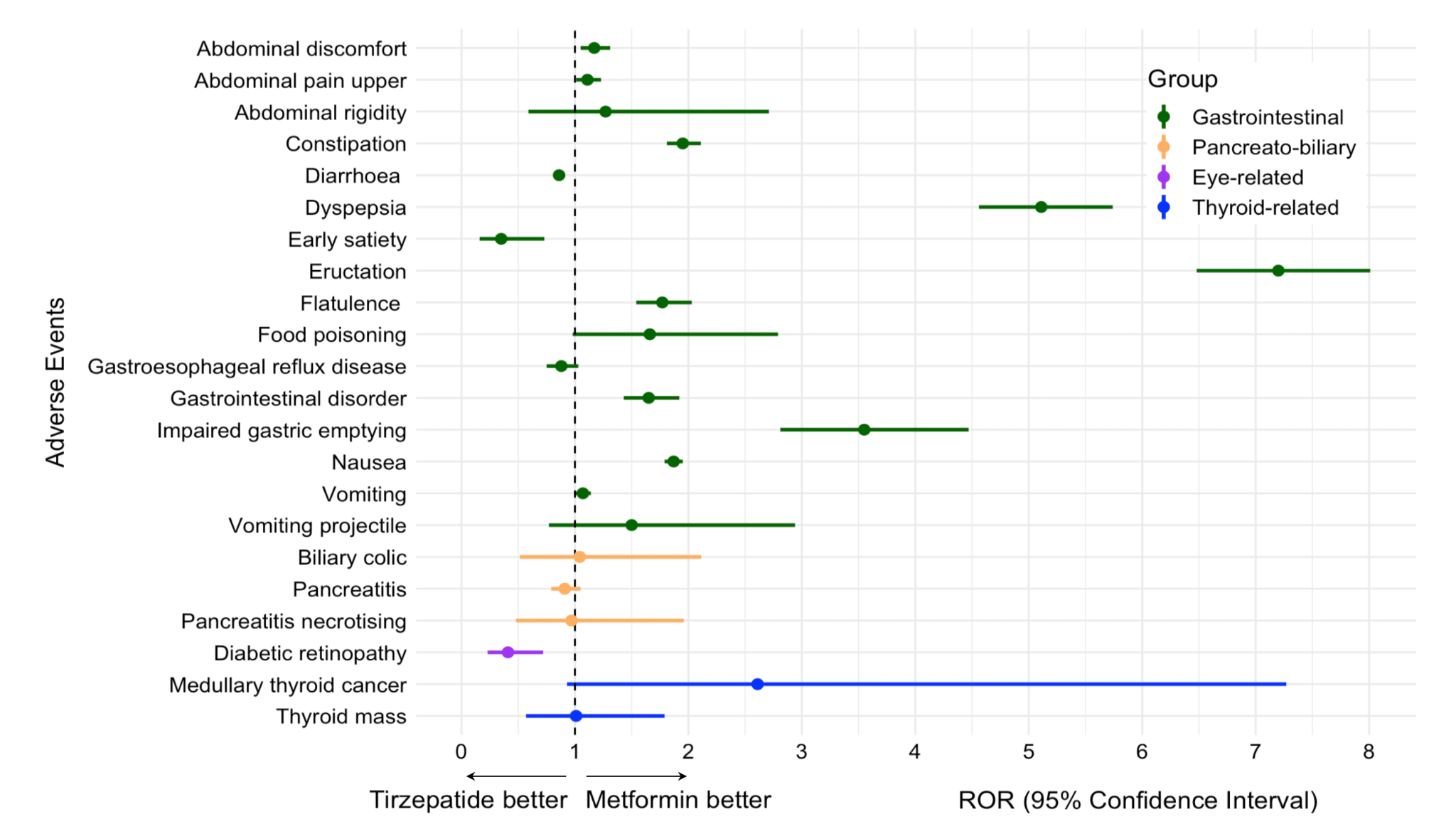
**

The forest plot shows reporting odds ratios (ROR) with 95% confidence intervals (CI) for gastrointestinal, pancreato-biliary, eye-related, and thyroid-related adverse events (AEs) for tirzepatide versus dulaglutide. A ROR <1.0 indicates a disproportional lower rate of AEs among reports for tirzepatide.
